# Web-Based Cognitive Behavioral Therapy Blended With Face-to-Face Sessions for Major Depression: Randomized Controlled Trial

**DOI:** 10.2196/10743

**Published:** 2018-09-21

**Authors:** Shigetsugu Nakao, Atsuo Nakagawa, Yoshiyo Oguchi, Dai Mitsuda, Noriko Kato, Yuko Nakagawa, Noriko Tamura, Yuka Kudo, Takayuki Abe, Mitsunori Hiyama, Satoru Iwashita, Yutaka Ono, Masaru Mimura

**Affiliations:** 1 Department of Neuropsychiatry Keio University School of Medicine Tokyo Japan; 2 Clinical and Translational Research Center Keio University Hospital Tokyo Japan; 3 National Center for Cognitive Behavior Therapy and Research National Center of Neurology and Psychiatry Kodaira Japan; 4 Department of Psychiatry National Hospital Organization Tokyo Medical Center Tokyo Japan; 5 Department of Psychiatry Sakuragaoka Memorial Hospital Tama Japan; 6 Department of Psychiatry St. Marianna University School of Medicine Kawasaki Japan; 7 Department of Preventive Medicine and Public Health Keio University School of Medicine Tokyo Japan; 8 Center for the Development of Cognitive Behavior Therapy Training Tokyo Japan

**Keywords:** blended cognitive behavioral therapy, cognitive behavioral therapy, major depressive disorder, major depression, randomized controlled trial

## Abstract

**Background:**

Meta-analyses of several randomized controlled trials have shown that cognitive behavioral therapy (CBT) has comparable efficacy to antidepressant medication, but therapist availability and cost-effectiveness is a problem.

**Objective:**

This study aimed to evaluate the effectiveness of Web-based CBT blended with face-to-face sessions that reduce therapist time in patients with major depression who were unresponsive to antidepressant medications.

**Methods:**

A 12-week, assessor-masked, parallel-group, waiting- list controlled, randomized trial was conducted at 3 medical institutions in Tokyo. Outpatients aged 20-65 years with a primary diagnosis of major depression who were taking ≥1 antidepressant medications at an adequate dose for ≥6 weeks and had a 17-item GRID-Hamilton Depression Rating Scale (HAMD) score of ≥14 were randomly assigned (1:1) to blended CBT or waiting-list groups using a computer allocation system, stratified by the study site with the minimization method, to balance age and baseline GRID-HAMD score. The CBT intervention was given in a combined format, comprising a Web-based program and 12 45-minute face-to-face sessions. Thus, across 12 weeks, a participant could receive up to 540 minutes of contact with a therapist, which is approximately two-thirds of the therapist contact time provided in the conventional CBT protocol, which typically provides 16 50-minute sessions. The primary outcome was the alleviation of depressive symptoms, as measured by a change in the total GRID-HAMD score from baseline (at randomization) to posttreatment (at 12 weeks). Moreover, in an exploratory analysis, we investigated whether the expected positive effects of the intervention were sustained during follow-up, 3 months after the posttreatment assessment. Analyses were performed on an intention-to-treat basis, and the primary outcome was analyzed using a mixed-effects model for repeated measures.

**Results:**

We randomized 40 participants to either blended CBT (n=20) or waiting-list (n=20) groups. All patients completed the 12-week treatment protocol and were included in the intention-to-treat analyses. Participants in the blended CBT group had significantly alleviated depressive symptoms at week 12, as shown by greater least squares mean changes in the GRID-HAMD score, than those in the waiting list group (−8.9 points vs −3.0 points; mean between-group difference=−5.95; 95% CI −9.53 to −2.37; *P*<.001). The follow-up effects within the blended CBT group, as measured by the GRID-HAMD score, were sustained at the 3-month follow-up (week 24) and posttreatment (week 12): posttreatment, 9.4 (SD 5.2), versus follow-up, 7.2 (SD 5.7); *P*=.009.

**Conclusions:**

Although our findings warrant confirmation in larger and longer term studies with active controls, these suggest that a combined form of CBT is effective in reducing depressive symptoms in patients with major depression who are unresponsive to antidepressant medications.

**Trial Registration:**

University Hospital Medical Information Network Clinical Trials Registry: UMIN000009242; https://upload.umin.ac.jp/cgi-open-bin/ctr_e/ctr_view.cgi?recptno=R000010852 (Archived by WebCite at http://www.webcitation. org/729VkpyYL)

## Introduction

Major depression is a common mental disorder with a serious public health impact. Over 300 million people globally are estimated to suffer from major depression, equivalent to 4.4% of the world’s population [1]. In addition, major depression is associated with increased morbidity and impaired function; it poses a significant societal and economic burden that accounts for 2.5% of the global disease burden [2]. Thus, it is predicted to be the leading cause of disability in high-income countries by 2030 and the third-leading cause in low-income and middle-income countries [3].

Meta-analyses of a large number of randomized controlled trials (RCTs) have shown that cognitive behavioral therapy (CBT) has efficacy comparable to antidepressant medication [4,5] and is more successful than antidepressant medication in reducing the risk of relapse after treatment ends [6,7]. In addition, the literature shows that many patients would like to access psychotherapy as an alternative or adjunct to pharmacotherapy [8,9]. Despite these compelling justifications for the widespread dissemination and implementation of CBT, significant barriers exist to providing CBT in the routine practice. One barrier to broadly disseminating CBT is an insufficient number of trained therapists [10]. The United Kingdom initiated a national project called “Improving Access to Psychological Therapies” to improve access to psychotherapy for patients with depression and anxiety. In this project, >3600 new therapists were trained and deployed in the initial 3 years; however, the program required a total cost of 309 million pounds (equivalent to US $405 million) [11]. Thus, large-scale CBT dissemination requires addressing inevitable resource allocation problems.

Computerized CBT (see Multimedia Appendix 1) is an alternative strategy for the broad dissemination of CBT and is considered to be more cost-effective. Although ample research has demonstrated the efficacy of computerized CBT in controlled research settings [12,13], the evidence to date indicates that computerized CBT without human support typically has much smaller effects and is associated with a higher rate of attrition than those with a modest amount of human support [12]. Furthermore, a pragmatic trial that tested the efficacy of 2 widely known computerized CBT programs delivered through a website, that is, Web-based CBT (the Mood Gym and Beating the Blues) with a small amount of telephone support in a primary care setting, found no clinical benefit and an extremely low treatment adherence [14].

To overcome low adherence while improving the beneficial effect of Web-based CBT, a newer treatment format, called blended CBT, where CBT sessions delivered by a therapist and computer are integrated into 1 treatment protocol, has been developed [15,16]; this blended format can be beneficial by tailoring sessions to meet patient-specific needs during therapist-delivered sessions, over and above the computerized program [17,18]. In addition, blended CBT aids in improving the efficiency by allowing therapists to focus more on process-related treatment components (eg, treatment introduction, evaluation, discussing thoughts and feelings, and asking questions about homework) in their therapist-delivered sessions, while more practical therapy components, such as psychoeducation, mood and activity diaries, and homework, can be done through the computerized program [18].

Despite the aforementioned advantages, so far only a few researchers have tested the efficacy of blended CBT in comparison with a control condition in the treatment of clinically diagnosed major depression. An 8-week blended CBT protocol developed by Wright et al [19] demonstrated beneficial effects compared with waiting list controls. Furthermore, a modified version of Wright’s earlier protocol, involving the therapy extension by another 8 weeks by adding 4 25-minute booster face-to-face sessions, showed similar effects as the conventional 16-week CBT protocol [20]. However, these trial participants were not currently taking antidepressant medications.

The blended CBT protocol used in this study was designed to integrate the Web-based CBT program using *Kokoro-no-skill-up-training* [21] with face-to-face sessions. *Kokoro-no*-*skill-up-training* (*Kokoro-no* means “for the mind” in Japanese) is a Web-based CBT program developed by one of our authors (YO), which provides computerized CBT modules. The stand-alone version of *Kokoro-no-skill-up-training* has demonstrated a beneficial effect on high-stress workers [22] and school students [23].

The objective of this study was to demonstrate that Web-based CBT blended with face-to-face sessions is effective in treating patients with major depression who are unresponsive to antidepressant medications, while reducing the therapist time. This study focuses on subjects with refractory depression because one-third of patients with major depression have considerable residual symptomatology after initial treatment [24,25]. Studies have shown that the addition of CBT is a promising strategy for refractory depression [26,27]. We, therefore, conducted an assessor-masked, 12-week, RCT to test the effectiveness of blended CBT for patients with major depression who did not respond to ≥1 antidepressant medication. Furthermore, in an uncontrolled explorative analysis, we investigated whether the expected positive effects of the intervention were sustained at follow-up, 3 months after the posttreatment assessment.

## Methods

### Design and Approval

This study was a 12-week, single-blind, waiting list controlled, randomized trial. The study was approved by the Ethics Committees of the study sites and registered in the University Hospital Medical Information Network Clinical Trials Registry (UMIN000009242). The study was conducted and reported in accordance with the Consolidated Standards of Reporting Trials of Electronic and Mobile HEalth Applications and onLine TeleHealth checklist [28].

### Participants

Participants were individuals who sought treatment for major depression at 3 study sites located in Tokyo: a university teaching hospital, a psychiatric hospital, and a general hospital. Those who agreed to participate were asked to provide written consent and undergo a baseline assessment.

Participants were eligible for inclusion in this study if they were aged 20-65 years and had Diagnostic and Statistical Manual of Mental Disorders, Fourth Edition (DSM-IV) major depressive disorder [29], as confirmed by the Structured Clinical Interview for DSM-IV-TR Axis I Disorders-Patient Edition (SCID-I/P) [30]. In addition, all participants met the operationalized criteria of having a 17-item GRID-Hamilton Depression Rating Scale (GRID-HAMD) [31,32] score of ≥14 despite having received adequate therapy with ≥1 antidepressant medications for at least 6 weeks as part of their routine care, and had access to the internet at home.

The exclusion criteria were a primary DSM-IV axis I diagnosis other than major depressive disorder, as assessed by the Mini-International Neuropsychiatric Interview [33,34], manic or psychotic episodes, alcohol or substance use disorder or antisocial personality disorder, serious and imminent suicidal ideation, organic brain lesions or major cognitive deficits, and serious or unstable medical illnesses. Moreover, those who had received CBT in the past (ie, defined as attending ≥8 CBT sessions) or who were unlikely to attend >8 sessions of study treatment (for reasons such as planned relocation) were excluded. The diagnostic interviews were conducted by treating psychiatrists, all of whom had received extensive training in the administration of semistructured interviews.

### Randomization and Masking

All eligible participants were randomly allocated (in a ratio of 1:1) to either the blended CBT group or the waiting list group. The allocation was concealed with the use of a Web-based random allocation system. Randomization was stratified by the study site using the minimization method to balance participants in terms of their age at study entry (<40 years, ≥40 years) and baseline GRID-HAMD score (14-18, ≥19).

Owing to the nature of interventions, although neither participants nor treating psychiatrists or therapists could be masked to the randomization status, the primary outcome measure (GRID-HAMD) was assessed by central assessors using the telephone. Central assessors were not involved with the treatment or study coordination and were prohibited from accessing any information that would reveal participant allocation. In addition, participants were instructed not to disclose their allocated treatment during telephonic interviews. All assessors were licensed clinical psychologists based at the National Center of Neurology and Psychiatry who had received extensive GRID-HAMD training and achieved excellent interrater reliability (intraclass correlation=.98). The percentage of agreement and kappa coefficients between the actual allocation and the allocation guessed by central assessors were 62.5% and .20 (95% CI –0.10 to 0.46) for the 6-week midpoint assessment and 60.0% and.25 (95% CI –0.06 to 0.52) for the 12-week posttreatment assessment, respectively, indicating that masking was successful.

### Treatment Procedures

#### Web-Based Cognitive Behavioral Therapy Blended With Face-to-Face Sessions

Participants allocated to the blended CBT arm were offered 12 weeks of Web-based CBT blended with 12 45-minute face-to-face sessions, with no booster session. Multimedia Appendix 2 provides an overview of the blended CBT program, which integrates the Web-based program using the *Kokoro-no-skill-up-training* with 12 weekly therapist sessions. The Web-based program consists of the following 5 core components: (1) psychoeducation, assessment, and problem clarification; (2) behavioral activation; (3) cognitive restructuring; (4) problem solving; and (5) relapse prevention (outline shown in Multimedia Appendix 3). By accessing the Web-based program, participants watched psychoeducational video clips and read short columns, rated and monitored their daily mood graphs, and mastered CBT skills, including behavioral activation, recognizing and addressing dysfunctional thoughts, and problem solving, by entering text as interactively guided on the Web screen. Although participants were encouraged to work with their Web-based program during the intervention period at their own pace, each module typically took about 30 minutes to complete. In the face-to-face session, therapists reviewed the material covered in the Web-based program, evaluated and discussed the participant-specific problem, and practiced CBT skills and set homework for the next session using the Web-based program. A guidebook was provided to facilitate mastery of the program. The guidebook offers a detailed session-by-session treatment procedure that includes information on how and when to use the specific Web-based content to meet the individualized needs of diverse participants.

We selected a 12-week format to improve the efficiency of treatment by reducing the number of therapist sessions compared with the standard Japanese CBT protocol, which offers 16 50-minutes sessions [35]. Thus, across 12 weeks, a participant could receive up to 540 minutes of contact with a therapist, approximately two-thirds of that provided in the conventional CBT protocol (ie, a total of 800 minutes). After 12 sessions of blended CBT, participants resumed usual care. Notably, 1 psychiatrist, 2 clinical psychologists with a doctoral degree, and 1 psychiatric nurse provided the blended CBT. Together, the therapists had practiced CBT for a mean of 6.0 (SD 2.4) years before the study. All therapists received CBT training, which included a 2-day intensive CBT workshop and 1-hour onsite group supervision every week from a skilled CBT supervisor (AN), with thorough reviews of cases and detailed feedback to maintain the adherence to CBT protocols and competence during the study. Participants allocated to the waiting list group also received the intervention after a 12-week waiting period and were informed about this before study entry.

#### Treatment as Usual

Participants allocated to both the blended CBT and waiting list groups continued treatment as usual with their treating psychiatrist. It consisted of medication management along with education regarding medication and dosage schedules, review of adverse effects, and supportive guidance from treating psychiatrists. Monitoring of depressive symptoms with the 16-item Quick Inventory of Depressive Symptomatology Self-Report (QIDS) [36,37] was conducted at each visit. Although there were no particular restrictions on the pharmacotherapy provided, treatments were in line with practice guidelines for depression care [38]. Three treating psychiatrists who had specialist experience in psychiatric care for a mean of 6.6 (SD 5.7) years provided the medication visits, which were offered roughly every 2 weeks, each visit lasting approximately 10 minutes. Notably, treating psychiatrists were not involved in the delivery or supervision of CBT.

### Outcomes

The primary outcome was the alleviation of depressive symptoms, as measured by the change in the total 17-item GRID-HAMD score from the baseline (at randomization: baseline assessment) to 12 weeks postrandomization (at the end of the intervention or waiting period: posttreatment assessment). The GRID-HAMD is an amended version of the original Hamilton Depression Rating Scale, which provides standardized explicit scoring conventions with a structured interview guide for administration and scoring [31,32]. In addition, changes were assessed after 6 weeks (midpoint assessment). Furthermore, outcome measures were assessed 3 months after the intervention was completed (follow-up assessment) in the blended CBT group only. For ethical reasons, we decided to offer CBT to participants from the control group after their participation in the waiting list study group. Therefore, the planned follow-up analysis to determine whether the effect of CBT was sustained was uncontrolled.

All secondary outcomes were also evaluated at the same time-points; these included treatment response (≥50% reduction in the baseline GRID-HAMD score); remission (GRID-HAMD score≤7); participant-rated measures of depressive symptoms, that is Beck Depression Inventory-Second Edition (BDI) score [39,40] and QIDS score; participant-rated inventory for depressogenic schemata, that is 24-item dysfunctional attitude scale (DAS-24) score [41,42]; and the quality-of-life status as measured by the mental and physical component summary score of the 36-Item Short-Form Health Survey [43,44]. Furthermore, participants were asked to complete the European Quality of Life Questionnaire-5 Dimensions to measure the health-related quality of life [45,46].

Information on the total daily dose of each antidepressant medication was expressed as a fraction of the World Health Organization’s defined daily dose [47], which is defined as the assumed average maintenance dose per day for adults calculated from the dosage recommendations for each drug. In addition, adverse events were monitored. Serious adverse events were defined as death, life-threatening events, events leading to severe impairment or dysfunction, and hospitalization.

### Statistical Analysis

Based on Wright et al [19], we assumed that a mean difference of 6 points on the 17-item GRID-HAMD score with an SD of 5.5 between the 2 groups would be clinically meaningful. With a two-sided 5% significance level and 90% power, a sample size of 18 was required for each group. Therefore, a total sample of 40 would provide sufficient power while also accounting for possible attrition. As we were not aware of any published blended CBT studies on refractory depression, we calculated the sample size of our study based on this prior study that included patients with a similar depression severity as our patients.

The primary analysis was performed on an intention-to-treat basis, and all randomized participants were included. For continuous outcomes, the least squares means (LS means) and their 95% CIs were estimated using a mixed-effects model for repeated measures (MMRM) for changes from the baseline, which contained the treatment group, week, and group-by-week interaction as fixed-effects with an unstructured covariance matrix among time-points; Kenward-Roger degrees of freedom adjustment were used. Mean changes for each group at each time-point and mean between-group differences were estimated using appropriate contrasts in the MMRM. Notably, missing values were not inputted. For categorical outcomes, relative risks (RRs) and their 95% CIs were calculated. The number needed to treat (NNT) was calculated when a 95% CI of RR did not include 1.0. In addition, we calculated the effect size (Cohen *d*) for all significant outcome measures (ie, as the difference in mean changes at posttreatment assessment between the blended CBT group and the waiting list group divided by the pooled SD of both groups). The effect size was classified as small (Cohen *d*=0.2-0.49), medium (Cohen *d*=0.5-0.79), or large (Cohen *d*>0.8) [48]. Furthermore, we investigated the follow-up effects in the blended CBT group with paired *t* tests, comparing the scores at follow-up assessment with the scores at posttreatment assessment. The significance level was set at.05 (two-tailed) for all analyses. No multiple-testing correction was applied because this study was an RCT with a single primary null-hypothesis. Statistical analyses were performed with SAS version 9.4 (SAS Institute, Cary, NC, USA).

## Results

**Figure 1:**
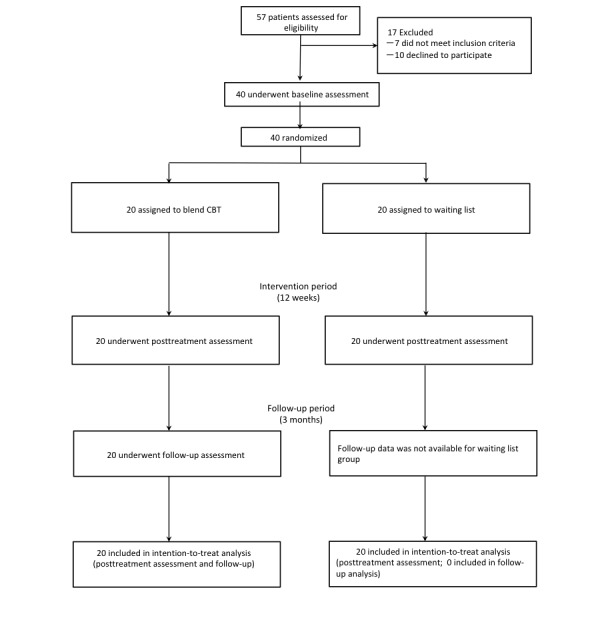
The Consolidated Standards of Reporting Trials diagram of participants’ flow through the study. CBT: cognitive behavioral therapy.

Figure 1 shows participants’ flow from screening to 3-months follow-up during the study. We screened 57 patients between November 29, 2011 and November 18, 2015, and the final 3-month follow-up was done on May 4, 2016. Of these 57 patients, 12% (7/57) did not meet the inclusion criteria and 18% (10/57) declined to participate. Therefore, 70% (40/57) eligible patients who agreed to participate and completed baseline assessments were randomized either to the blended CBT (n=20) or waiting list (n=20) group. There were no dropouts from the study, and all participants allocated to the blended CBT group completed the follow-up assessment.

Table 1 summarizes the sociodemographic and clinical characteristics of participants at the baseline. The mean age of participants was 40.2 (SD 9.8) years, and the percentage of males was 50% (20/40). Furthermore, 68% (27/40) participants had received one course of antidepressant medication and 33% (13/40) had received 2 courses before study entry. None of the participants had received >3 courses of antidepressant medication before study entry.

Tables 2 and 3 show treatment engagement by the study groups.

**Table 1:** Characteristics of participants at the baseline.

Characteristic		Blended CBT^a^ (n=20)	Waiting list (n=20)	Total (n=40)
Age (years), mean (SD)		39.7 (9.4)	40.6 (10.2)	40.2 (9.7)
Male, n (%)		10 (50)	10 (50)	20 (50)
Education (years), mean (SD)		15.4 (1.9)	15.6 (2.6)	15.4 (2.2)
Unemployed, n (%)		3 (15)	4 (20)	7 (18)
**Marital status, n (%)**				
	Married	8 (40)	10 (50)	18 (45)
	Separated, divorced, or widowed	2 (10)	0 (0)	2 (5)
	Single (never married)	10 (50)	10 (50)	20 (50)
Cohabiting, n (%)		14 (70)	16 (80)	30 (75)
Number of lifetime depression episodes, mean (SD)		1.5 (0.6)	1.7 (1.4)	1.6 (1.1)
History of psychiatric hospitalization, n (%)		1 (5)	3 (15)	4 (10)
Prior suicide attempt, n (%)		1 (5)	1 (5)	2 (5)
Self-reported childhood abuse, n (%)		2 (10)	5 (25)	7 (18)
Self-reported victims of childhood bullying, n (%)		5 (25)	3 (15)	8 (20)
Family history of psychiatric disorders, n (%)		7 (35)	5 (25)	12 (30)
Duration of index depression episode (months), mean (SD)		27.3 (29.8)	20.3 (32.3)	23.8 (30.9)
**Particulars of index episode (DSM-IV^b^), n (%)**				
	Chronic (≥2 years of index episode)	8 (40)	4 (20)	12 (30)
	Melancholic features	12 (60)	11 (55)	23 (58)
	Atypical features	0 (0)	0 (0)	0 (0)
**Comorbid DSM-IV Axis I diagnoses, n (%)**				
	Any anxiety disorder	1 (5)	5 (25)	6 (15)
	Panic disorder (with or without agoraphobia)	0 (0)	2 (10)	2 (5)
	Social anxiety disorder	1 (5)	1 (5)	2 (5)
	Obsessive compulsive disorder	0 (0)	0 (0)	0 (0)
	Generalized anxiety disorder	0 (0)	0 (0)	0 (0)
	Dysthymic disorder	0 (0)	0 (0)	0 (0)
**Number of prior courses of antidepressant treatment, n (%)**				
	1-2 courses	15 (75)	15 (75)	30 (75)
	3-4 courses	2 (10)	3 (15)	5 (13)
	5-6 courses	2 (10)	1 (5)	3 (8)
	7-10 courses	1 (5)	0 (0)	1 (3)
	>10 courses	0 (0)	1 (5)	1 (3)
**Number of antidepressant medications prescribed at baseline, n (%)**				
	1 medication	14 (70)	13 (65)	27 (67.5)
	≥2 medications	6 (30)	7 (35)	13 (32.5)
**Health-related quality of life, mean (SD)**				
	European Quality of Life Questionnaire 5-dimension, mean (SD)	0.7 (0.1)	0.8 (0.1)	0.7 (0.1)
**Depression severity**				
	17-item GRID-Hamilton Depression Rating Scale score, mean (SD)	18.3 (3.7)	18.5 (3.6)	18.4 (3.6)
	Beck Depression Inventory-Second Edition score, mean (SD)	28 (8.8)	24.4 (7.8)	26.2 (8.4)
	Quick Inventory of Depressive Symptomatology Self-Report score, mean (SD)	14.8 (4.2)	13.5 (4)	14.1 (4.1)

^a^CBT: cognitive behavioral therapy.

^b^DSM-IV: Diagnostic and Statistical Manual of Mental Disorders, Fourth Edition.

**Table 2:** Treatment engagement of the study group (n=20).

Treatment engagement	Value
Mean number of blended CBT^a^ sessions attended, mean (SD)	11.65 (2.32)
Completion rate of the full course of blended CBT sessions (*n* of blended CBT completers/*n* of blended CBT participants), n (%)	20 (100)
Mean duration of face-to-face-CBT sessions (minutes), mean (SD)	44.3 (6.85)
Mean duration of medication visits (minutes, over 12-weeks), mean (SD)	11.6 (2.3)

^a^CBT: cognitive behavioral therapy.

**Table 3:** Treatment engagement of the groups.

Treatment engagement		Blended CBT^a^ (n=20)	Waiting list (n=20)	*P* value^b^
Mean medication compliance over 12-weeks, treatment and medication compliance data scale self-report, n (%)		19 (97)	19 (96)	.67
Number of medication visits over 12-weeks, mean (SD)		8 (1)	7 (1)	.37
**Mean antidepressant medication dose at baseline and 12 weeks, mean (SD)**				
	0 week (baseline)	1.39 (0.58)	1.31 (0.75)	.74
	12 weeks	1.21 (0.68)	1.31 (0.75)	.67
**Changes in antidepressant prescription by the end of the 12-week intervention period, n (%)**				
	No change	14 (70)	10 (50)	.20
	Switched to another antidepressant	2 (10)	1 (5)	>.99
	Increased antidepressant dose	1 (5)	1 (5)	>.99
	Combined another antidepressant	1 (5)	3 (15)	.61
	Decreased	0 (0)	3 (15)	.23
	Stopped antidepressant	2 (10)	2 (10)	>.99

^a^CBT: cognitive behavioral therapy.

^b^*P* values are for *t* test for continuous outcomes and chi-square test for categorical outcomes.

All participants allocated to the blended CBT group completed the full program course (defined as attending at least 8 sessions). The therapist adherence (yes or no) to the CBT treatment protocol was at high level—100% (n/allocators=20/20) for behavioral activation component, 90% (18/20) for cognitive reconstruction component, and 85% (17/20) for problem-solving component. In terms of the medication management, the mean daily dose of antidepressant medications was comparable at each assessment point between the groups, and no significant dose changes were observed during the 12-week interventional period. Selective serotonin reuptake inhibitors were the most common antidepressant medication prescribed at the baseline, 35% (14/40) participants (Multimedia Appendix 4). There were no differences in the number of medical visits between the groups.

**Figure 2:**
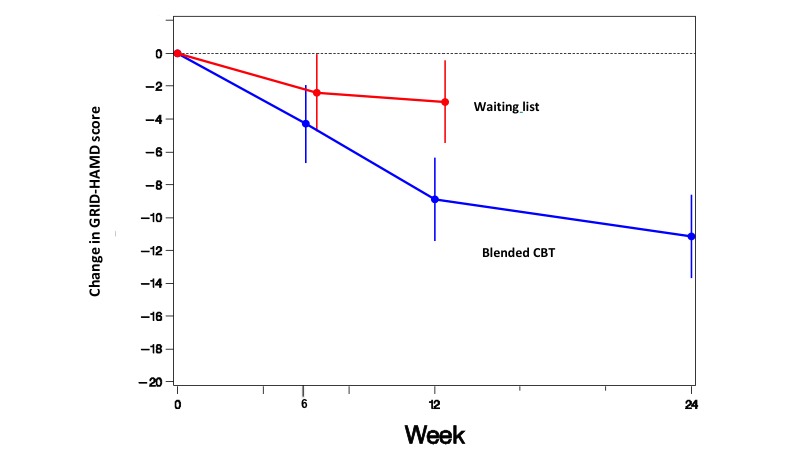
Least squares mean change in the 17-item GRID-Hamilton Depression Rating Scale (GRID-HAMD) score over time in participants allocated to the blended cognitive behavioral therapy (CBT) group or waiting list group.

Participants in the blended CBT group had significantly alleviated depressive symptoms after 12 weeks, as shown by greater LS mean changes in the GRID-HAMD score compared with that in the waiting list group (−8.9 points vs −3.0 points; mean between-group difference=−5.95; 95% CI −9.53 to −2.37; *P*=.0002; Cohen *d*=1.0; Figure 2). Figure 2 shows LS mean changes and their 95% CIs in the GRID-HAMD total scores over time estimated with a MMRM analysis; error bars indicate 95% CIs. In addition, the follow-up effects showed that the GRID-HAMD score at 3-month follow-up had improved significantly compared with the GRID-HAMD score after 12 weeks: 9.4 (SD 5.2) vs 7.2 (SD 5.7); *P*=.009. Of note, no significant treatment effect was observed after 6 weeks (*P*=.25).

Tables 4 and 5 summarize the secondary outcome measures. Participants allocated to the blended CBT group were 2.8 times more likely to have a treatment response at the posttreatment (12 weeks) assessment than those in the waiting list group (RR, 2.75; 95% CI 1.05-7.20), resulting in an NNT of 3 (95% CI 1.6-14.2). In addition, the blended CBT group demonstrated achievement of significant remission at the posttreatment assessment (12 weeks; RR, 8.00, 95% CI 1.10-58.19; NNT: 3, 95% CI 1.7-8.7). Although the blended CBT group participants had milder depressive symptoms, as measured by the BDI, than the participants of the waiting-list group over the intervention period, differences were not statistically significant at each assessment point. There were no statistically significant differences between the groups in the intensity of depressogenic schemata, as assessed by the DAS-24, and in the quality-of-life status, as assessed by the 36-Item Short-Form Health Survey, mental and physical subscales, and the European Quality of Life Questionnaire-5 Dimensions, over the study period. Of note, none of the participants experienced serious adverse events during the intervention period.

**Table 4:** Summary of repeated measures analyses of outcomes: treatment response and remission (intention-to-treat population).

Timepoint of achieving response or remission		Blended CBT^a^ (n=20), n (%)	Waiting list (n=20), n (%)	Comparison	
				Relative risks (95% CI)	*P* value
**Response (≥50% reduction in 17-item GRID-HAMD^b^)**					
	6 weeks	2 (10)	2 (10)	1.00 (0.16 to 6.42)	>.99
	12 weeks	11 (55)	4 (20)	2.75 (1.05 to 7.20)	.04
	24 weeks (3-month follow-up) only CBT	15 (75)	N/A^c^	N/A	N/A
**Remission (GRID-HAMD≤7)**					
	6 weeks	2 (10)	1 (5)	2.00 (0.20 to 20.33)	.56
	12 weeks	8 (40)	1 (5)	8.00 (1.10 to 58.19)	.04
	24 weeks (3-month follow-up) only CBT	13 (65)	N/A	N/A	N/A
**Response (≥50% reduction in QIDS^d^)**					
	6 weeks	12 (60)	6 (30)	2.00 (0.94 to −4.27)	.07
	12 weeks	9 (45)	3 (15)	3.00 (0.95 to −9.48)	.06
	24 weeks (3-month follow-up) only CBT	14 (70)	N/A	N/A	N/A
**Remission (QIDS≤5)**					
	6 weeks	6 (30)	4 (20)	1.50 (0.20 to −20.33)	.56
	12 weeks	6 (30)	3 (15)	2.00 (0.58 to −6.91)	.27
	24 weeks (3-month follow-up) only CBT	11 (55)	N/A	N/A	N/A
**Response (≥50% reduction in BDI^e^)**					
	6 weeks	3 (15)	2 (10)	1.50 (0.28 to −8.04)	.64
	12 weeks	7 (35)	3 (15)	2.33 (0.70 to −7.76)	.17
	24 weeks (3-month follow-up) only CBT	10 (50)	N/A	N/A	N/A
**Remission (BDI≤13)**					
	6 weeks	4 (20)	5 (25)	0.80 (0.25 to −2.55)	.71
	12 weeks	8 (40)	5 (25)	1.60 (0.63 to −4.05)	.32
	24 weeks (3-month follow-up) only CBT	10 (50)	N/A	N/A	N/A

^a^CBT: cognitive behavioral therapy.

^b^GRID-HAM: GRID-Hamilton Depression Rating Scale.

^c^N/A: not applicable.

^d^QIDS: Quick Inventory of Depressive Symptomatology.

^e^BDI: Beck Depression Inventory-Second Edition.

**Table 5:** Summary of repeated measures analyses of outcomes: participant-rated measures (intention-to-treat population).

Participant-rated measures		Blended CBT^a^, mean (SD)	Waiting list, mean (SD)	Difference in mean change scores^b,c^ (95% CI)	*P* value
**17-item GRID-Hamilton Depression Rating Scale score**					
	0 week (baseline)	18.3 (3.6)	18.5 (3.6)	N/A^d^	N/A
	6 weeks	14.0 (5.7)	16.1 (4.7)	1.90 (−1.46 to 5.26)	.26
	12 weeks	9.4 (5.1)	15.5 (6.3)	5.95 (2.37 to 9.53)	.002
	24 weeks (3-month follow-up) only CBT	7.2 (5.7)	N/A	N/A	N/A
**Beck Depression Inventory-Second Edition score**					
	0 week (baseline)	28.0 (8.6)	24.4 (7.6)	N/A	N/A
	6 weeks	23.4 (11.2)	20.1 (8.2)	0.35 (−4.63 to 5.33)	.89
	12 weeks	18.5 (12.3)	20.8 (9.1)	5.85 (−0.27 to 11.97)	.06
	24 weeks (3-month follow-up) only CBT	14.7 (12.5)	N/A	N/A	N/A
**Quick Inventory of Depressive Symptomatology Self-Report score**					
	0 week (baseline)	14.8 (4.0)	13.5 (3.9)	N/A	N/A
	6 weeks	7.9 (3.7)	8.7 (3.8)	2.20 (−0.66 to 5.06)	.13
	12 weeks	8.0 (4.7)	10.2 (4.2)	3.55 (0.53 to 6.57)	.02
	24 weeks (3-month follow-up) only CBT	6.8 (5.5)	N/A	N/A	N/A
**Dysfunctional attitude scale score**					
	0 week (baseline)	97.1 (19.0)	87.6 (17.1)	N/A	N/A
	6 weeks	97.4 (19.2)	86.1 (17.8)	−1.80 (−9.23 to 5.63)	.63
	12 weeks	92.4 (23.6)	84.9 (18.5)	2.05 (−6.82 to 10.92)	.64
	24 weeks (3-month follow-up) only CBT	83.4 (21.8)	N/A	N/A	N/A
**European Quality of Life Questionnaire-5 Dimensions score**					
	0 week (baseline)	0.7 (0.1)	0.7 (0.1)	N/A	N/A
	6 weeks	0.8 (0.1)	0.8 (0.1)	−0.04 (−0.12 to 0.04)	.29
	12 weeks	0.8 (0.1)	0.8 (0.1)	−0.07 (−0.16 to 0.01)	.08
	24 weeks (3-month follow-up) only CBT	0.8 (0.1)	N/A	N/A	N/A
**36-item Short-Form Health Survey (SF-36) mental component summary score**					
	0 week (baseline)	37.7 (10.6)	37.8 (7.7)	N/A	N/A
	6 weeks	39.5 (9.7)	41.6 (8.3)	1.96 (−3.45 to 7.37)	.47
	12 weeks	43.9 (10.2)	41.3 (7.9)	−2.70 (−8.91 to 3.50)	.38
	24 weeks (3-month follow-up) only CBT	44.6 (11.1)	N/A	N/A	N/A
**SF-36 physical component summary score**					
	0 week (baseline)	52.8 (11.2)	51.7 (10.8)	N/A	N/A
	6 weeks	50.8 (11.0)	53.5 (7.4)	3.87 (−1.90 to 9.64)	.18
	12 weeks	49.4 (13.5)	53.4 (10.6)	5.04 (−2.81 to 12.89)	.20
	24 weeks (3-month follow-up) only CBT	52.8 (9.6)	N/A	N/A	N/A

^a^CBT: cognitive behavioral therapy.

^b^The difference in the mean change in scores is the intergroup difference in the least squares mean treatment change score from the baseline to the data point, from the mixed-effects model for repeated measures analysis.

^c^The intergroup difference is the CBT group value minus the waiting list group value.

^d^N/A: not applicable.

## Discussion

This study tested the effectiveness of the blended CBT program in patients with major depression who did not respond to antidepressant treatment after taking ≥1 antidepressant medications at adequate doses for ≥6 weeks, compared with waiting list control conditions. The between-group effect size after blended CBT was large (Cohen *d*=1.0), and these results were similar to Wright et al’s blended CBT trial using waiting list controls (Cohen *d*=1.14) [19]. In addition, we were able to reduce the therapist contact time by about two-thirds compared with the standard CBT protocol. Furthermore, we focused on patients who were unresponsive to antidepressant medications.

There are several possible reasons for the high level of treatment protocol adherence with no dropouts with the blended CBT program in this study. First, the program had a blended format rather than a stand-alone Web-based CBT program. Patients might have developed a stronger treatment engagement through the therapist-delivered session because of tailoring the program according to patient-specific needs and may have gained mastery of CBT skills by accessing the interactive Web-based program. Second, owing to the shortage of trained therapists [49] and insufficient health insurance coverage of CBT sessions in Japan (health insurance does not cover CBT sessions delivered by psychologists), participants may have had strong expectations from CBT. Dunlop et al reported that patients matched to their preferred treatment could achieve a higher rate of treatment completion than those who were mismatched [50].

Significant alleviation of depressive symptoms was found in the assessment of primary (GRID-HAMD) and secondary outcome measures of depressive symptoms (QIDS). Furthermore, the remission rate (8/20, 40%) as measured by the GRID-HAMD was similar to that reported by Thase et al [20]. In contrast to their findings, our study did not show significant differences in self-reported depressive symptomatology, as measured by the BDI and DAS-24, between the groups. As this study was powered to detect the effectiveness based on the primary outcome measure, it is possible that these secondary outcome measures were underpowered. In addition, the BDI and DAS-24 baseline scores of our sample were lower than those reported by Thase et al [20], which could have been because of a floor effect.

Our blended CBT program has a unique format, integrating Web-based and offline components back to back. Patients engage themselves in a self-directed Web-based learning module, such as by reading psychoeducational columns and watching video clips, before coming to the face-to-face session. Moreover, in the subsequent face-to-face session, patients assimilate and apply what they have learned in the Web-based component to reinforce mastery of CBT skills. Thus, this blended format appears to correspond with the newer educational system, known as “blend learning” or “inverted classroom,” which is reported to be more effective than purely face-to-face or purely Web-based classes [51]. In addition, this blended format is perhaps promising for trainee therapists. With the Web-based assistance, therapists can deliver CBT techniques with more confidence, despite little experience. In fact, studies have shown that computer-assisted training is effective in training clinicians in empirically supported manual-guided therapies [52,53].

This study has several limitations. First, this study used waiting list controls rather than active controls. The use of the waiting list group could have provoked nocebo effects [54,55]. However, all our participants allocated to the waiting list group continued their usual treatment with their psychiatrists, and the course of depression symptoms was similar to the course of patients receiving psychiatric care [25]. Second, the benefits of blended CBT observed in this study cannot be solely attributed to the intervention because there was no treatment control. In other words, nonspecific treatment effects, such as patient expectations, may also account for the observed efficacy of the intervention. Nevertheless, this study aimed to examine the effectiveness of blended CBT rather than evaluating the effects of blended CBT itself. The third possible limitation is that this study was of relatively small size, although the number of participants exceeded that required for power analysis. Fourth, participants were recruited from 3 sites with a zero dropout rate, suggesting a cohort of highly motivated treatment-seeking patients, which may limit generalizability. Finally, there was no control group during the follow-up phase. Hence, a sustained effect during this phase cannot be attributed with certainty to the effects of acute therapy with our blended CBT program.

In conclusion, this study suggests that our blended CBT program was effective in reducing depressive symptoms in patients with major depression compared with waiting list controls. Additional research is now needed to replicate our results with larger sample size, longer observation period, and using active controls before definite conclusions can be drawn.

## Appendix

Multimedia Appendix 1 Modes of delivery of computerized cognitive behavioral therapy.

Multimedia Appendix 2 Overview of the blended cognitive behavioral therapy (CBT) program.

Multimedia Appendix 3 The framework of the 12-week web-based cognitive behavioral therapy blended with face-to-face sessions for major depression.

Multimedia Appendix 4 Type and dose of antidepressant medication prescribed at baseline.

Multimedia Appendix 5 CONSORT‐EHEALTH checklist (V 1.6.1).

## Abbreviations

BDI: Beck Depression Inventory-Second Edition
CBT: cognitive behavioral therapy
DAS: dysfunctional attitude scale
DSM-IV: Diagnostic and Statistical Manual of Mental Disorders, Fourth Edition
GRID-HAMD: GRID-Hamilton Depression Rating Scale
LS: least squares
MMRM: mixed-effects model for repeated measures
NNT: number needed to treat
QIDS: Quick Inventory of Depressive Symptomatology Self-Report
RR: relative risk
