# Supplementary material for: Web-Based Cognitive Behavioral Therapy Blended With Face-to-Face Sessions for Major Depression: Randomized Controlled Trial
Source: J Med Internet Res. 2018 Sep 21;20(9):e10743. doi: 10.2196/10743 (PMC6231848; doi:10.2196/10743)
Supplement: Multimedia Appendix 1 [file jmir_v20i9e10743_app1.pdf]

Multimedia Appendix 1

Modes of delivery of computerized cognitive behavioral therapy

Computerized cognitive behavioral therapy (CCBT) is a generic term for the delivery of CBT via an interactive computer interface delivered by a personal computer, internet, or interactive voice response system. Early versions of CCBT were delivered offline using CD-ROMs and were subsequently adapted for web-based delivery.

- **Computer-based CBT**
  CBT program delivered using offline electronic products, such as CD-ROMs.
  Examples: Beating the Blues CD-ROM package (UK), Good Day Ahead-The multimedia program for cognitive therapy DVD-ROM package (US).

- **Internet-based CBT**
  CBT program delivered using non-web based internet components, such as emails or video chat.
  Examples: Out of depression; DAVID (Sweden) [1].

- **Web-based CBT**
  CBT delivered using a web-based interactive program. Web-based CBTs are often offered without therapist guidance (unguided interventions), but can be combined with minimal, albeit regular, therapist contact (therapist-guided interventions).
  Examples: Mood GYM (Australia) [2], Beating the Blues (UK) [3], Deprexis (Germany) [4], Wellbeing program (Australia) [5].

- **Blended CBT**
  Blended CBT is a relatively new format where online and face-to-face interventions are intergraded into one treatment protocol.
  Examples: Blended CBT combining web-based Good Day Ahead program and face-to-face sessions (US) [6].

CONFIDENTIAL

REFERENCES

1. Andersson G, Bergstrom J, Hollandare F, Carlbring P, Kaldo V, Ekselius L. Internet-based self-help for depression: randomised controlled trial. Br J Psychiatry. 2005 Nov;187:456-61. PMID: 16260822. doi: 10.1192/bjp.187.5.456.
2. Christensen H, Griffiths KM, Korten A. Web-based cognitive behavior therapy: analysis of site usage and changes in depression and anxiety scores. J Med Internet Res. 2002 Jan-Mar;4(1):e3. PMID: 11956035. doi: 10.2196/jmir.4.1.e3.
3. Proudfoot J, Ryden C, Everitt B, Shapiro DA, Goldberg D, Mann A, et al. Clinical efficacy

of computerised cognitive-behavioural therapy for anxiety and depression in primary care: randomised controlled trial. Br J Psychiatry. 2004 Jul;185:46-54. PMID: 15231555.

4. Meyer B, Berger T, Caspar F, Beevers CG, Andersson G, Weiss M. Effectiveness of a novel integrative online treatment for depression (Deprexis): randomized controlled trial. J Med Internet Res. 2009 May 11;11(2):e15. PMID: 19632969. doi: 10.2196/jmir.1151.
5. Titov N, Andrews G, Davies M, McIntyre K, Robinson E, Solley K. Internet treatment for depression: a randomized controlled trial comparing clinician vs. technician assistance. PLoS One. 2010 Jun 8;5(6):e10939. PMID: 20544030. doi: 10.1371/journal.pone.0010939.
6. Wright JH, Wright AS, Albano AM, Basco MR, Goldsmith LJ, Raffield T, et al. Computer-assisted cognitive therapy for depression: maintaining efficacy while reducing therapist time. Am J Psychiatry. 2005 Jun;162(6):1158-64. PMID: 15930065. doi: 10.1176/appi.ajp.162.6.1158

CONFIDENTIAL
