# Supplementary material for: Web-Based Cognitive Behavioral Therapy Blended With Face-to-Face Sessions for Major Depression: Randomized Controlled Trial
Source: J Med Internet Res. 2018 Sep 21;20(9):e10743. doi: 10.2196/10743 (PMC6231848; doi:10.2196/10743)
Supplement: Multimedia Appendix 2 [file jmir_v20i9e10743_app2.pptx]

## Slide 1
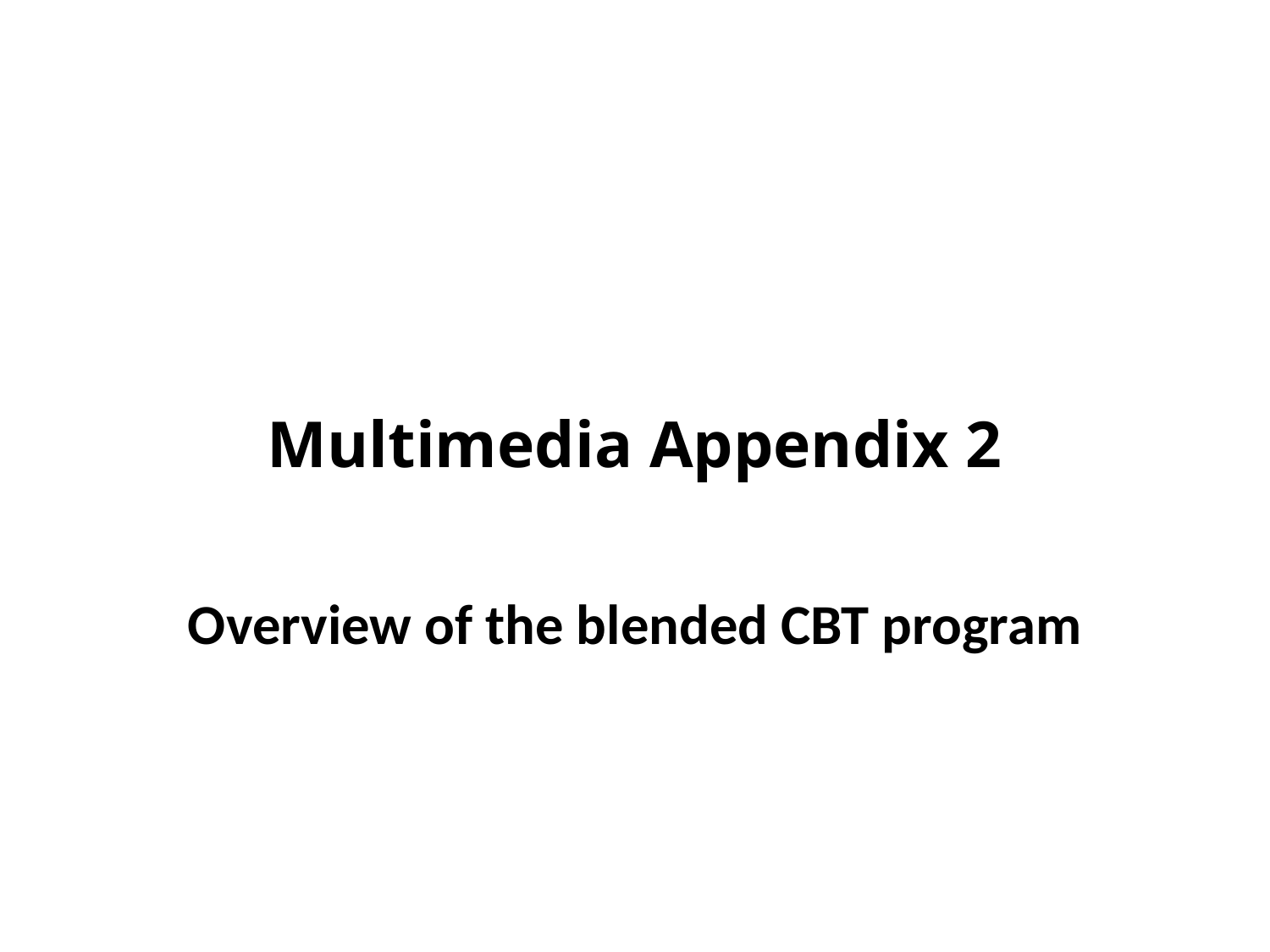

# Multimedia Appendix 2
Overview of the blended CBT program

## Slide 2
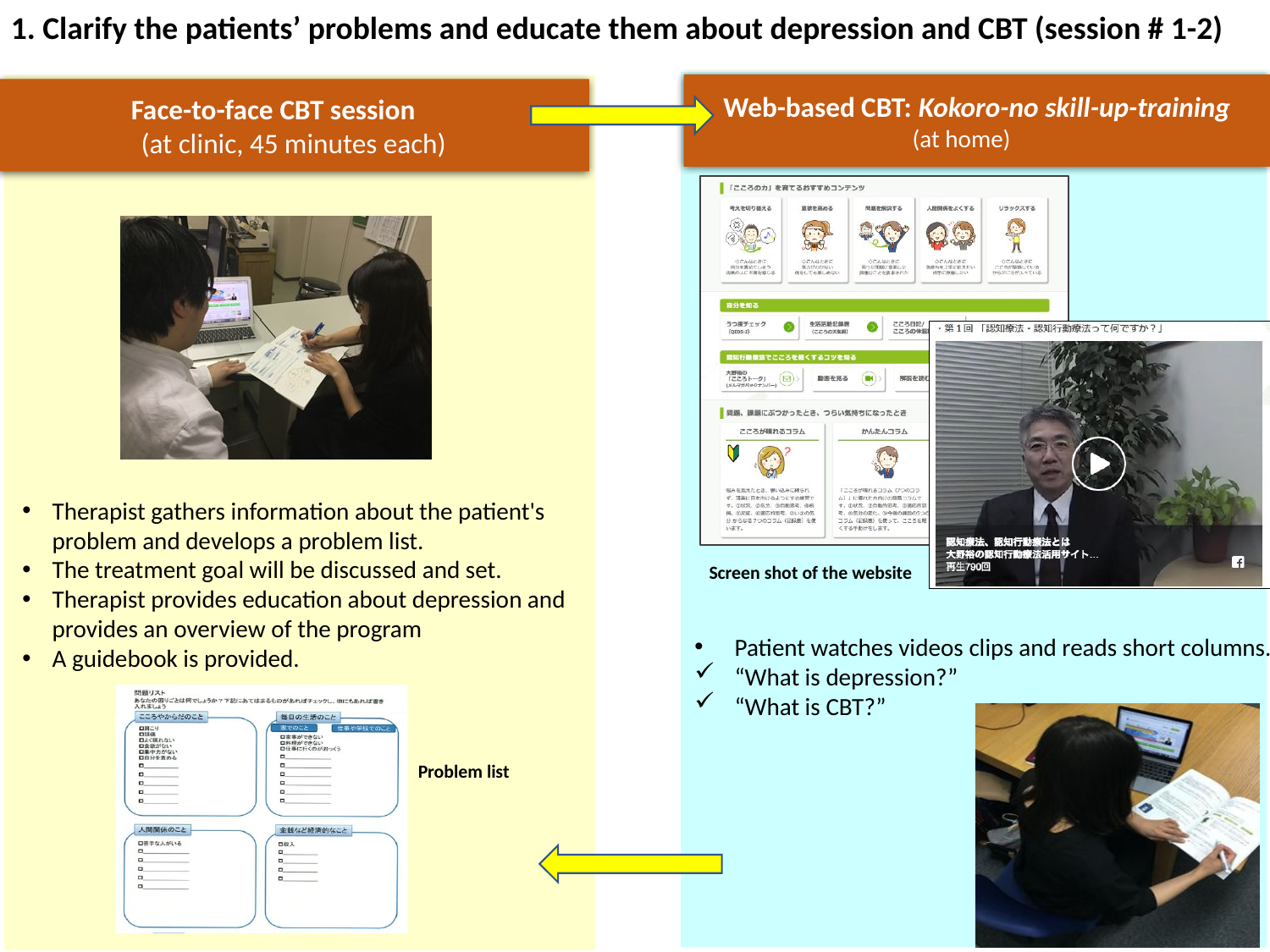

1. Clarify the patients’ problems and educate them about depression and CBT (session # 1-2)
	‘What is depression?’
	‘What is CBT?
Web-based CBT: Kokoro-no skill-up-training
(at home)
Face-to-face CBT session
(at clinic, 45 minutes each)
Therapist gathers information about the patient's problem and develops a problem list.
The treatment goal will be discussed and set.
Therapist provides education about depression and provides an overview of the program
A guidebook is provided.
Screen shot of the website
Patient watches videos clips and reads short columns.
“What is depression?”
“What is CBT?”
Problem list

## Slide 3
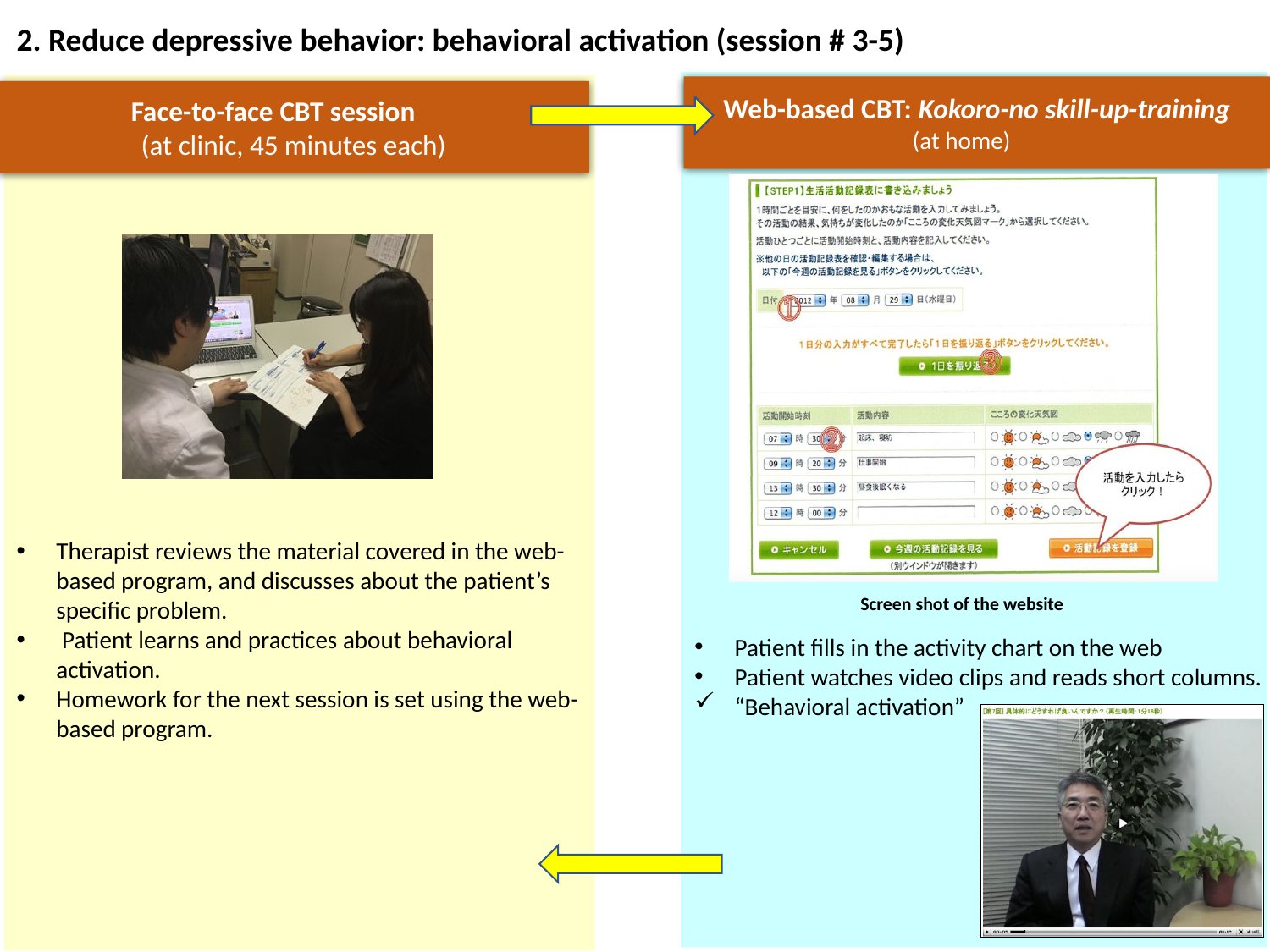

2. Reduce depressive behavior: behavioral activation (session # 3-5)
	‘What is depression?’
	‘What is CBT?
Web-based CBT: Kokoro-no skill-up-training
(at home)
Face-to-face CBT session
(at clinic, 45 minutes each)
Therapist reviews the material covered in the web-based program, and discusses about the patient’s specific problem.
 Patient learns and practices about behavioral activation.
Homework for the next session is set using the web-based program.
Screen shot of the website
Patient fills in the activity chart on the web
Patient watches video clips and reads short columns.
“Behavioral activation”

## Slide 4
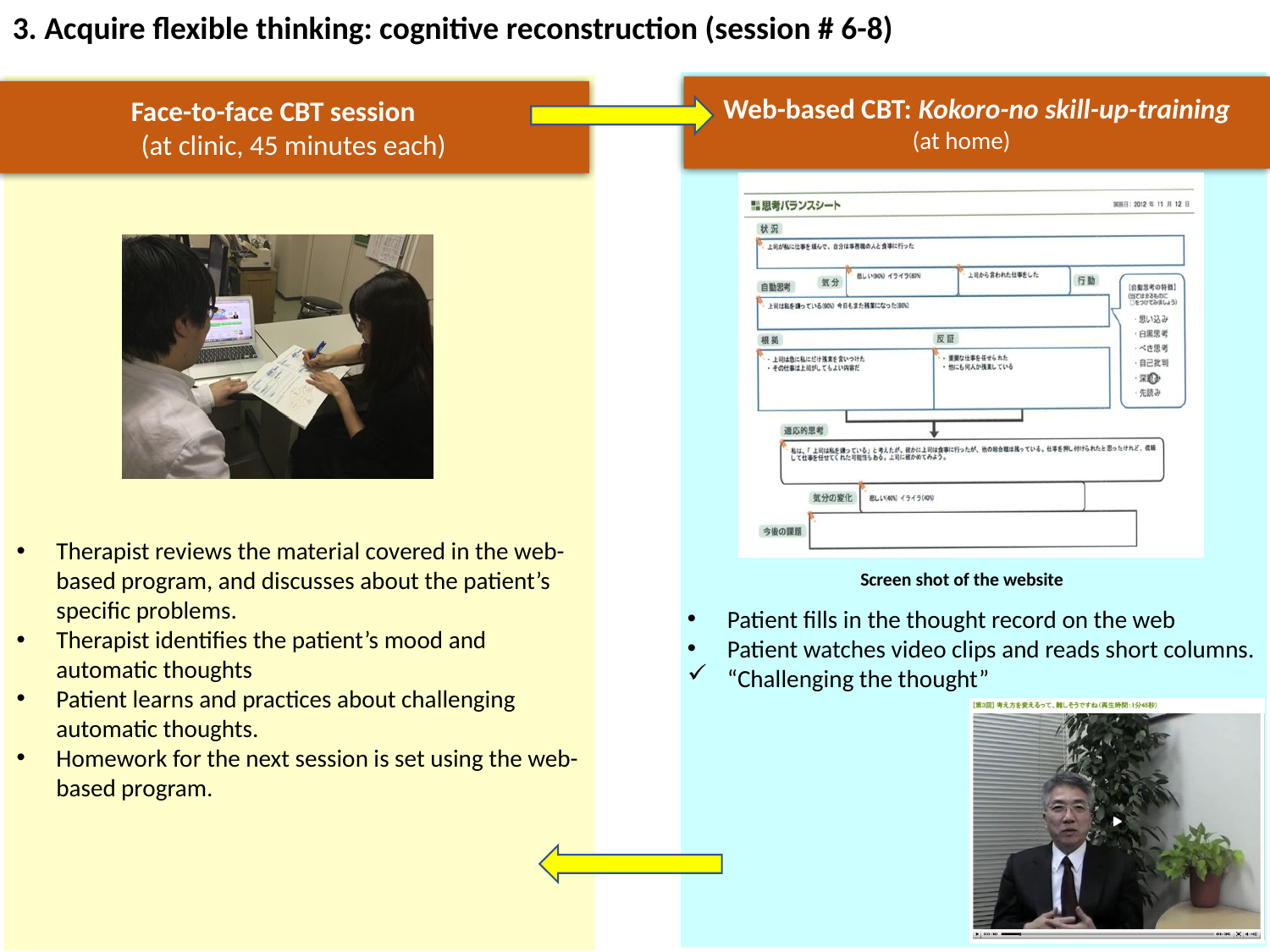

3. Acquire flexible thinking: cognitive reconstruction (session # 6-8)
Web-based CBT: Kokoro-no skill-up-training
(at home)
Face-to-face CBT session
(at clinic, 45 minutes each)
Therapist reviews the material covered in the web-based program, and discusses about the patient’s specific problems.
Therapist identifies the patient’s mood and automatic thoughts
Patient learns and practices about challenging automatic thoughts.
Homework for the next session is set using the web-based program.
Screen shot of the website
Patient fills in the thought record on the web
Patient watches video clips and reads short columns.
“Challenging the thought”

## Slide 5
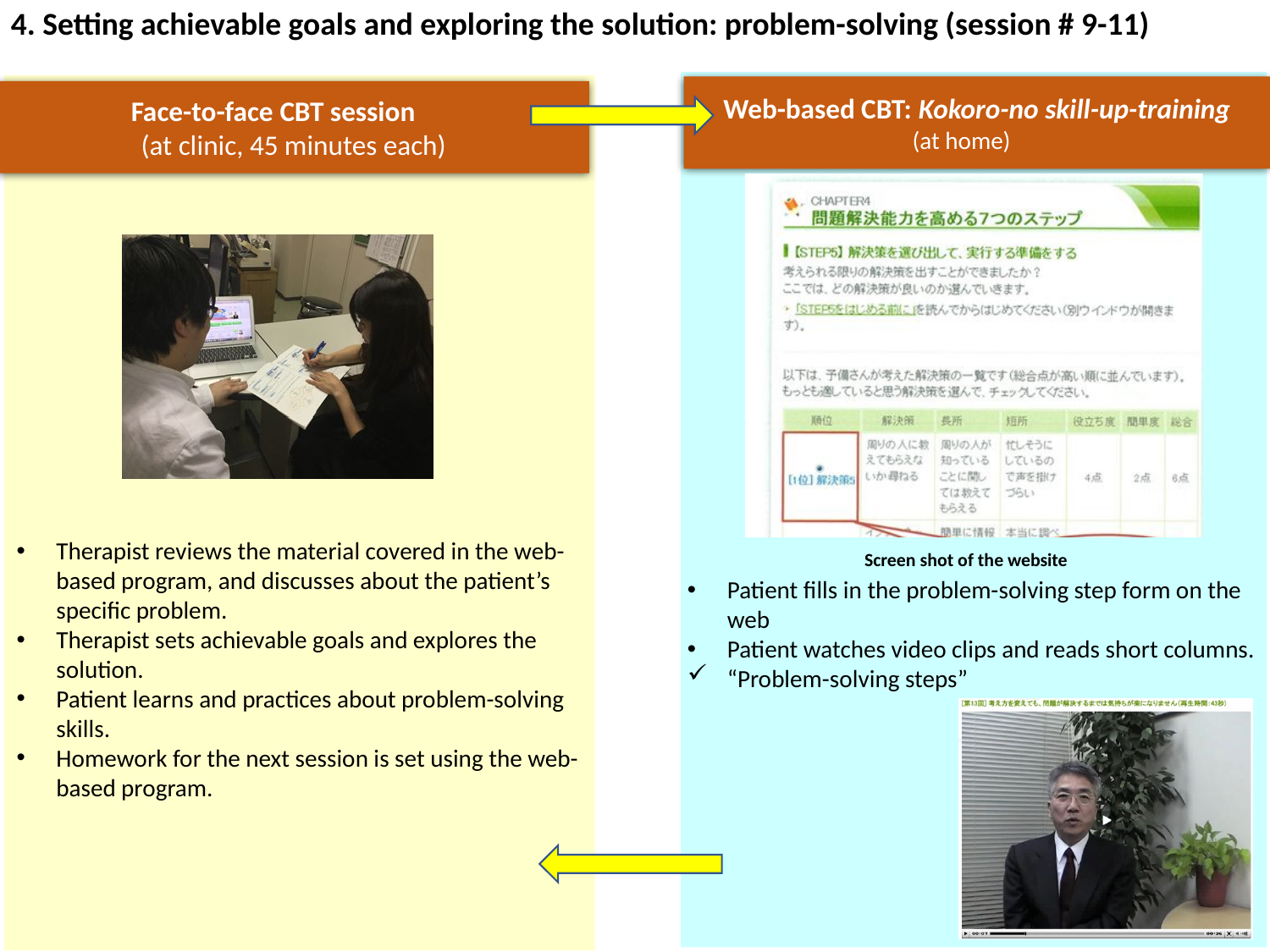

4. Setting achievable goals and exploring the solution: problem-solving (session # 9-11)
Web-based CBT: Kokoro-no skill-up-training
(at home)
Face-to-face CBT session
(at clinic, 45 minutes each)
Therapist reviews the material covered in the web-based program, and discusses about the patient’s specific problem.
Therapist sets achievable goals and explores the solution.
Patient learns and practices about problem-solving skills.
Homework for the next session is set using the web-based program.
Screen shot of the website
Patient fills in the problem-solving step form on the web
Patient watches video clips and reads short columns.
“Problem-solving steps”

## Slide 6
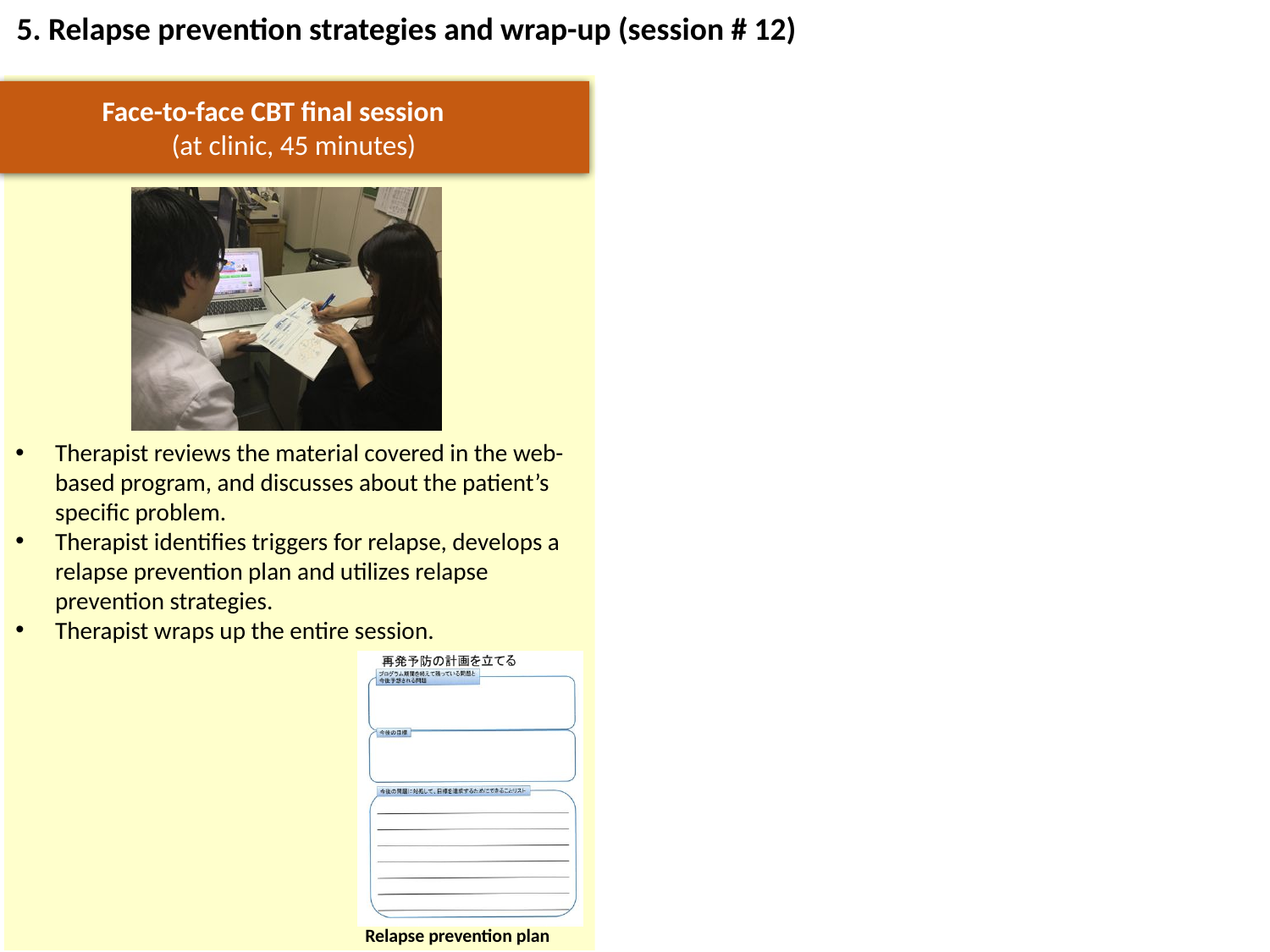

5. Relapse prevention strategies and wrap-up (session # 12)
Face-to-face CBT final session
(at clinic, 45 minutes)
Therapist reviews the material covered in the web-based program, and discusses about the patient’s specific problem.
Therapist identifies triggers for relapse, develops a relapse prevention plan and utilizes relapse prevention strategies.
Therapist wraps up the entire session.
Relapse prevention plan
