# Supplementary material for: Web-Based Cognitive Behavioral Therapy Blended With Face-to-Face Sessions for Major Depression: Randomized Controlled Trial
Source: J Med Internet Res. 2018 Sep 21;20(9):e10743. doi: 10.2196/10743 (PMC6231848; doi:10.2196/10743)
Supplement: Multimedia Appendix 3 [file jmir_v20i9e10743_app3.pdf]

Multimedia Appendix 3

The framework of the 12-week web-based cognitive behavioral therapy blended with face-to-face sessions for major depression

Session #	Suggested structure	Goals of the face-to-face session	Suggested website module /Homework
1,2	✓ Review symptoms, course of the illness and developmental history<br>✓ Clarify patient's problems<br>✓ The treatment goal will be discussed and set.<br>✓ Educate patient about depression and CBT<br>✓ Provide a summary and elicit feedback	✓ Establish rapport<br>✓ Gather information about the patient's problems and develop a problem list<br>✓ Provide education about depression and an overview of the program	✓ Read the guidebook<br>✓ "What is depression?"<br>✓ "What is CBT?"
3,4,5	✓ Understand the interaction between behavior and mood<br>✓ Reduce depressive behavior: behavioral activation	✓ Learn and practice about behavioral activation	✓ "Behavioral activation"<br>✓ Fill in the activity chart
6,7,8	✓ Understand dysfunctional thoughts<br>✓ Create a dysfunctional thought record<br>✓ Acquire flexible thinking: cognitive reconstructing	✓ Identify mood and automatic thoughts<br>✓ Challenge automatic thoughts	✓ "Challenging the thought"<br>✓ Fill in the three-column thought record<br>✓ Fill in the seven-column thought record
9,10,11	✓ Define problems<br>✓ Setting achievable goals and exploring the solution: problem-solving	✓ Learn problem-solving skills	✓ "Problem-solving steps“<br>✓ Fill in the problem-solving step form
12	✓ Identify triggers for relapse, develop a relapse prevention plan and utilize relapse prevention strategies<br>✓ Wrap-up	✓ Highlight relapse prevention<br>✓ Termination	
