# Supplementary material for: Web-Based Cognitive Behavioral Therapy Blended With Face-to-Face Sessions for Major Depression: Randomized Controlled Trial
Source: J Med Internet Res. 2018 Sep 21;20(9):e10743. doi: 10.2196/10743 (PMC6231848; doi:10.2196/10743)
Supplement: Multimedia Appendix 4 [file jmir_v20i9e10743_app4.pdf]

Multimedia Appendix 4

Type and dose of antidepressant medication prescribed at baseline

Antidepressant medication		Dose (mg)	Blended CBT (n= 20)	Waiting-list (n= 20)
SSRI total			10 (50.0%)	4 (20.0%)
Sertraline		50-100	7 (35.0%)	2 (10.0%)
Paroxetine		40	1 (5.0%)	1 (5.0%)
Fluvoxamine		50	1 (5.0%)	0 (0.0%)
Escitalopram		20	1 (5.0%)	1 (5.0%)
SNRI total			5 (25.0%)	6 (30.0%)
Duloxetine		40-60	5 (25.0%)	6 (30.0%)
TCA total			4 (20.0%)	2 (10.0%)
Amoxapine		100-150	1 (5.0%)	2 (10.0%)
Clomipramine		50	1 (5.0%)	0 (0.0%)
Nortriptyline		100-150	2 (10.0%)	0 (0.0%)
Other total			1 (5.0%)	8 (40.0%)
Mirtazapine		15-45	1 (5.0%)	6 (30.0%)
Sulpiride		100	0 (0.0%)	1 (5.0%)
Trazodone		25	0 (0.0%)	1 (5.0%)
**Medication used for combination/augmentation therapy[a]**		**Dose (mg)**	**Blended CBT (n=20)**	**Waiting-list (n=20)**
Sertraline	SSRI	25	1 (5.0%)	1 (5.0%)
Paroxetine	SSRI	20	0 (0.0%)	1 (5.0%)
Duloxetine	SNRI	20-30	3 (15.0%)	0 (0.0%)
Mirtazapine	Other	15-45	1 (5.0%)	3 (15.0%)
Sulpiride	Other	100	1 (5.0%)	1 (5.0%)
Trazodone	Other	25	1 (5.0%)	0 (0.0%)
Aripiprazole	SGA	3-6	1 (5.0%)	1 (5.0%)
Olanzapine	SGA	2.5	0 (0.0%)	2 (10.0%)
Perospirone	SGA	16	0 (0.0%)	1 (5.0%)
Quetiapine	SGA	12.5-25	0 (0.0%)	2 (10.0%)
Risperidone	SGA	1	1 (5.0%)	0 (0.0%)
Chlorpromazine	FGA	12.5	1 (5.0%)	0 (0.0%)
Levomepromazine	FGA	5	0 (0.0%)	1 (5.0%)
Lithium	Mood stabilizer	600	0 (0.0%)	2 (10.0%)
Lamotrigine	Mood stabilizer	200	0 (0.0%)	1 (5.0%)

[a] Some patients received more than two medications as part of combination/augmentation therapy.
